# Informative Missingness in Nominal Data: A Graph-Theoretic Approach to Revealing Hidden Structure

**DOI:** 10.34133/csbj.0099

**Published:** 2026-05-18

**Authors:** Ehsan Zangene, Veit Schwämmle, Mohieddin Jafari

**Affiliations:** ^1^ Department of Pharmacology, Faculty of Medicine, University of Helsinki, Helsinki, Finland.; ^2^ Department of Biochemistry and Molecular Biology, University of Southern Denmark, Odense, Denmark.; ^3^ Faculty of Medicine and Health Technology, Tampere University, Tampere, Finland.; ^4^ Tampere Institute for Advanced Study, Tampere University, Tampere, Finland.

## Abstract

Missing data is often treated as a nuisance, routinely imputed or excluded from statistical analyses, especially in nominal datasets where its structure cannot be easily modeled. However, the form of missingness itself can reveal hidden relationships, substructures, and biological or operational constraints within a dataset. In this study, we present a graph-theoretic approach that reinterprets missing values not as gaps to be filled, but as informative signals. By representing nominal variables as nodes and encoding observed or missing associations as edges, we construct both weighted and unweighted bipartite graphs to analyze modularity, nestedness, and projection-based similarities. This framework enables downstream clustering and structural characterization of nominal data based on the topology of observed and missing associations; edge prediction via multiple imputation strategies is included as an optional downstream analysis to evaluate how well inferred values preserve the structure identified in the nonmissing data. Across a series of biological, ecological, and social case studies, including proteomics data, the BeatAML drug screening dataset, ecological pollination networks, and human resource analytics, we demonstrate that the structure of missing values can be highly informative. These configurations often reflect meaningful constraints and latent substructures, providing signals that help distinguish between data missing at random and not at random. When analyzed with appropriate graph-based tools, these patterns can be leveraged to improve the structural understanding of data and provide complementary signals for downstream tasks such as clustering and similarity analysis. Our findings support a conceptual shift: Missing values are not merely analytical obstacles but valuable sources of insight that, when properly modeled, can enrich our understanding of complex systems across domains.

## Graphical Abstract

**Figure:**
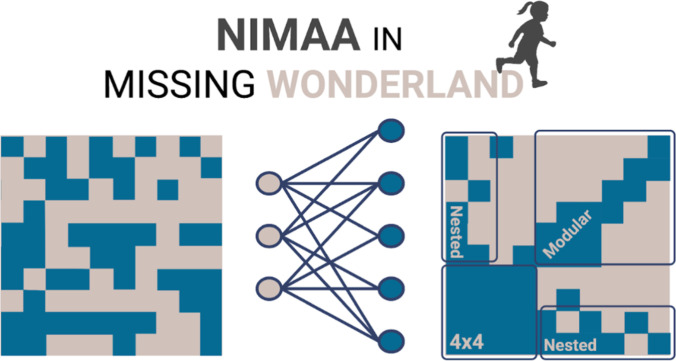

## Introduction

Missing values in datasets are often considered problematic artifacts that need to be corrected or removed through imputation [1–3]. This approach can be effective, especially when we understand their origin and can distinguish between missing at random (MAR) and missing not at random (MNAR) values [1,2,4]. Here, informative missingness refers to missing values whose arrangement is itself meaningful, because it reflects underlying structure in the data rather than only random absence. Missing data mechanisms are commonly categorized as missing completely at random (MCAR), where missingness is independent of both observed and unobserved data; MAR, where missingness depends only on observed variables; and MNAR, where missingness depends on unobserved values themselves [5]. In practice, distinguishing between these mechanisms can be challenging, and structured missingness patterns may reflect a combination of these processes [6].

However, a careful examination of missingness patterns or forms can itself provide meaningful structural insights [7,8]. In this context, we define informative missingness as the presence of nonrandom topological patterns in the arrangement of missing edges within the bipartite network, such as modularity, nestedness, or clustering, which deviate from expectations under random missingness. This is particularly important for nominal data, which consists of multiple categorical labels without inherent numeric or ordinal relationships.

Such data are prevalent across biomedicine, social sciences, and ecology [9–11], where they are used to classify observations into discrete groups (e.g., patient IDs, drug names, protein accessions, cell types, and species). In cancer research, for example, such nominal representations have been used to uncover novel dependencies and therapeutic strategies [12]. Because nominal data lack intrinsic order or magnitude, they offer more limited analytical flexibility than ordinal or numerical data [12]. While they can be incorporated into conventional statistical methods [13,14] this typically requires transformation (e.g., encoding) and restricts the use of common summary measures such as the mean or median. In addition, missing values introduce further challenges for interpretation and analysis [12,15]. In other words, the common methods often overlook the potentially informative form of missing values, which may reflect underlying biological variability, data collection constraints, or system-level organization. This limitation highlights a critical need for frameworks that can extract information from the structure of observed and missing nominal label associations without making assumptions about the causes of missingness.

The idea that missing values can carry information has been explored previously, particularly in the context of time-series data and supervised learning frameworks, where informative missingness is modeled as an explicit feature for prediction. For example, missing patterns have been leveraged to improve classification performance in longitudinal datasets [16]. In contrast, NomInal data Mining AnAlysis (NIMAA) focuses on nominal data, where labels lack intrinsic order or magnitude and standard feature-based modeling is not directly applicable. Our contribution lies in a graph-theoretic treatment of missingness, where the topology and arrangement of absent associations in bipartite networks are analyzed directly, without relying on supervised labels or temporal structure. The growing interest in network-based modeling, particularly within precision medicine, has led to the development of numerous tools for analyzing complex interactions among biological entities [17].

**Fig. 1.**
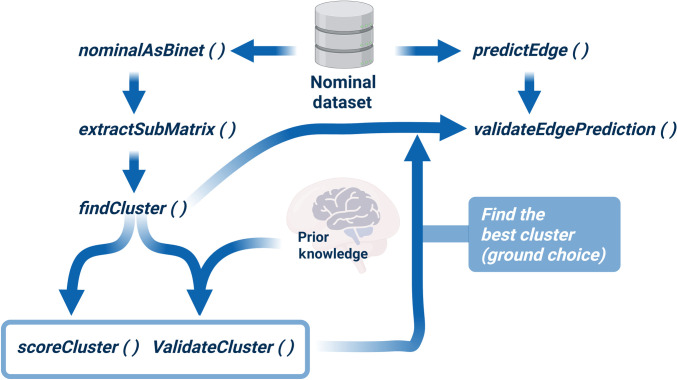
Overview flowchart of the NomInal data Mining AnAlysis (NIMAA) pipeline and its main analytical steps. Starting from nominal data, the workflow constructs an incidence matrix, extracts complete submatrices, performs projection-based clustering and validation, and optionally applies edge imputation followed by structural comparison of imputed and nonmissing networks.

Here, we develop a nominal data mining approach based on bipartite network modeling to explore hidden present and missing relationships among the nominal labels. Using 9 datasets spanning biological and social domains, we demonstrate how patterns of missingness can influence the interpretation of the data and the underlying systems they represent. We also present this framework in the NIMAA R Shiny application built on the NIMAA R package [18], an open-source toolkit that provides functions for constructing bipartite networks from nominal data, analyzing missingness structure (e.g., nestedness, clustering, and projection), and optionally performing imputation and validation within a reproducible workflow. The major functions of the NIMAA pipeline are summarized in the overview flowchart shown in Fig. 1.

Briefly, the workflow begins with plotIncMatrix(), which constructs and visualizes the incidence matrix. Complete submatrices can then be extracted using extractSubMatrix() for downstream analyses on nonmissing regions. Projection-based clustering is performed using findCluster(), while scoreCluster() and validateCluster() provide internal and external validation of clustering results. As an optional downstream step, predictEdge() applies multiple imputation strategies to estimate missing values, and validateEdgePrediction() evaluates how well the imputed networks preserve the structural patterns identified in the nonmissing data. This step enables the evaluation and comparison of imputation strategies based on their ability to preserve the structural properties identified in the nonmissing data, thereby providing a practical framework for researchers interested in downstream network completion and validation.

Several existing approaches, including stochastic block models, biclustering algorithms, and graph-based link prediction methods, aim to infer latent structure or predict missing edges based on observed interactions. In contrast, NIMAA focuses on the topology of missingness itself, treating the arrangement of absent edges as a source of structural information. By analyzing patterns such as modularity, nestedness, and clustering within missing entries prior to imputation, the framework provides a complementary perspective that emphasizes data structure exploration before predictive modeling. While we provide an accompanying R package and Shiny interface for practical implementation, the primary contribution of this work is the underlying methodological framework for analyzing structured missingness in nominal data.

## Methods and Implementation

To provide a theoretical overview of our approach to nominal data mining using the NIMAA pipeline, consider a dataset ${\left\{\left(x_{i},\;y_{i}\right)\right\}}_{i=1}^{n}$ where $x_{i}\in V\wedge y_{i}\in W$ are nominal variables. This dataset can be represented as a bipartite network in which *V* and *W* are disjoint node sets of the network, and each observation $\left(x_{i},\;y_{i}\right)$ represents a link between corresponding nodes. If each link has an associated measurement, the network becomes weighted. The connectivity structure is represented by a biadjacency (or incidence) matrix A. Two projections of the network can be taken, one for V and one for W. The adjacency matrix for the projection on the part V is $A\times A^{T}$ where $A^{T}$ is the transpose of the matrix A. The adjacency matrix of the projection on the part W can also be defined using $A^{T}\times A$. In the projected unipartite networks, the edge weight between 2 nodes equals the sum of products of their connections to nodes in the opposite set. This means that the edge weight counts the sum of the product of edge weights if the bipartite network is weighted. To facilitate the analysis of relationships within each set of entities, the bipartite graph can be projected into a unipartite graph. In this projection, nodes from one set (e.g., rows) are connected if they share common associations with nodes from the other set (e.g., columns). This projection approach allows us to implement network analysis techniques for the unipartite to bipartite case. Since these projections capture pairwise similarities within each node set, community detection on the projected networks reveals groups of similar nodes.

Beyond basic similarity and clustering analysis, bipartite networks often exhibit higher-order structural patterns. Two key examples are nestedness and modularity. Nestedness, defined as the tendency for nodes to interact with subsets of the interaction partners of better-connected nodes, is one of the important structural patterns identified in bipartite networks [19]. Nestedness describes a hierarchical ordering of nodes in which more specialized nodes interact with a subset of the partners with whom more generalized nodes interact (see the graphical abstract). It is common to rank rows and columns of the incidence matrix to visualize nestedness. As a result, the matrix, which is typically displayed in the lattice format of filled and empty squares, presents a decreasing connectivity with a large number of connections packed in the upper left corner. The most popular nestedness estimator is the “temperature” of Atmar and Patterson [20]. A median line in the ranked incidence matrix is used to create this index. This line divides the matrix into holes and full cells equally (zeros and ones). The “temperature” index quantifies the dispersion of holes in relation to the median. In contrast to nestedness, which reflects hierarchical interaction patterns, modularity captures the presence of distinct communities within the network. Modularity identifies a network’s community structure as distinct clusters of interactions (see the graphical abstract), with more connections within communities than between communities [21]. To ensure meaningful structural analysis, we first compute nestedness and modularity indices and then address missing data by identifying and isolating complete submatrices within the incidence matrix. We might be interested in extracting the nonmissing part of a weighted bipartite network. In this case, we rearrange the incidence matrix to find an approximation of the complete submatrix that includes nonmissing values. It should be noted that for a given matrix, it is nondeterministic polynomial-time (NP)-hard to find the “largest” column- or row-wise submatrix without any missing value [22]. In NIMAA, missing values (NA [not available]) are treated as unknown interactions rather than true zeros; the absence of an observed edge does not imply confirmed noninteraction. Understanding the structure of missing values in the dataset is essential, as some patterns may suggest that certain NAs correspond to true zeros, while others reflect unobserved or incomplete measurements.

Additionally, when complete submatrices are insufficient for downstream analysis, we apply imputation methods, such as mean, median, and correspondence analysis (CA), to estimate missing values. These imputed values enable us to generate optimized network projections (see below). These networks show the similarity between each node in each part of the bipartite network. Clustering analysis is used to explore the structural pattern of similarities between nodes. When we have prior knowledge about nodes, we compare clustering results with node labels based on prior knowledge. The Jaccard and Rand indices, for example, are used to measure the similarity between 2 clusterings of data. The following section outlines the implementation steps of the NIMAA pipeline and illustrates its application using results from 9 diverse datasets analyzed with this methodology. In general, structurally oriented analysis is more meaningful when missingness shows systematic dependence on observed or unobserved data (e.g., MAR or MNAR), whereas MCAR patterns are less likely to contain interpretable structural information.

The computational performance of the NIMAA framework depends on the size and sparsity of the bipartite incidence matrix, as well as the selected downstream analyses. Core steps, such as incidence matrix construction and visualization, scale approximately linearly with the number of observed edges, while projection, clustering, and imputation steps may introduce higher computational costs depending on the algorithms used. As many nominal datasets are inherently sparse, the framework is generally efficient for moderate to large datasets. For very large datasets, performance can be improved through standard strategies such as filtering low-frequency nodes, analyzing subgraphs, or selecting computationally efficient clustering methods.

### Global structure of the data and submatrix extraction with nonmissing values

For a dataset containing 2 nominal variables, NIMAA constructs a bipartite network. The input data typically consists of a table with 2 columns representing the nominal variables and an optional third column containing numerical values that serve as edge weights. This information is transformed into an incidence matrix using the plotIncMatrix() function. The incidence matrix is visualized as a heatmap, highlighting both observed and missing values while also capturing structural properties such as modularity and nestedness within the bipartite network. This visualization provides a complementary perspective on the relationships between categories and further illustrates the forms of missingness within the network structure. The resulting matrix object can then be passed to the plotBipartite() function, which generates an interactive igraph-based bipartite network plot, offering a complementary perspective on the relationships and missingness patterns in the data.

In the next step, we extract a complete submatrix containing only nonmissing values from the weighted bipartite network using the extractSubMatrix() function. This function rearranges the original matrix to identify a large, densely connected rectangular region without missing entries. The function employs an iterative heuristic that repeatedly reorders rows and columns based on individual element values as well as row and column sums to approximate a maximal complete submatrix. It is important to note that identifying the true maximal submatrix without missing values is an NP-hard problem; therefore, the function provides an efficient approximation rather than an exact solution. In practice, multiple similarly dense submatrices may exist, and small variations in the data can lead to alternative but qualitatively similar solutions. The extracted submatrix enables more reliable downstream analyses by focusing on coherent data blocks with fully observed relationships. For further details on the implementation, we refer the reader to the NIMAA package vignette.

### Analysis of projected networks

As mentioned above, 2 projected networks are constructed from a bipartite network. The clustering analysis of the projected networks identifies the cluster of similar nodes in each vertex set based on the similarity of neighbors of each node in the bipartite network. The weight of the edges in a weighted bipartite network affects pairwise similarity. With the option to preprocess the input incidence matrix, the findCluster() function generates projected unipartite networks and then applies 7 network clustering algorithms. These include walktrap, multilevel, infomap, label propagation, leading eigenvector, spinglass, and fast greedy community detection methods. Weak edges can also be removed before clustering. Then, based on internal or external cluster validation indices such as the average silhouette width and Jaccard similarity index, the best clustering method can be chosen. The results are offered as bar plots in addition to a console display. Two external validation indices, the Jaccard similarity coefficient and Rand index, are also provided to measure the similarity between clustering results and the prior knowledge used as ground truth if prior knowledge on the label of nodes is included in the dataset.

### Bipartite edge prediction by weight imputation

The predictEdge() function provides an optional downstream analysis step that applies multiple imputation strategies to assess the robustness of structural patterns identified prior to imputation. Rather than treating imputed values as ground truth, this step evaluates whether inferred edges preserve clustering and similarity relationships derived from complete submatrices. Supported techniques include classical approaches such as mean and median imputation, as well as CA [23] and alternating least squares [24]. These methods were selected to represent a range of imputation strategies with different levels of complexity, including simple baseline approaches (mean and median) for reference, and structure-aware methods (CA and alternating least squares) that are compatible with matrix factorization and bipartite network representations, while remaining computationally efficient for moderate-sized datasets. Once imputation is performed, the resulting completed matrices are projected into unipartite networks for downstream clustering. To evaluate the impact of imputation, the validateEdgePrediction() function compares clustering results obtained from imputed networks against those derived from the maximal submatrix of nonmissing values (serving as a benchmark). Several similarity and validation metrics, including the Sørensen–Dice coefficient, Fowlkes–Mallows index, Jaccard similarity, Minkowski index, and Rand index, are computed to quantify the consistency of clustering outcomes [25]. By integrating these metrics, NIMAA enables the systematic selection of the most reliable imputation method, ensuring that edge predictions preserve the underlying structure and relationships within the original nominal dataset. It should be noted that this evaluation focuses on the preservation of structural patterns rather than reconstruction accuracy and therefore complements, rather than replaces, conventional validation approaches based on masked data experiments or error metrics such as root mean square error.

The validation strategy implemented in validateEdgePrediction() relies on comparisons within complete submatrices, where ground truth is available. While this enables assessment of structural consistency (e.g., preservation of clustering patterns), it may not fully capture imputation performance in sparse or missing regions, particularly under MNAR mechanisms where missingness is systematically related to unobserved values. As a result, validation outcomes should be interpreted as indicative of performance in well-observed regions and not as a guarantee of accuracy in unobserved or structurally missing parts of the data.

## Case Studies

In this section, we present 9 datasets grouped into 4 major thematic categories: proteomic data benchmarking, biomedical/pharmacological associations, ecological/environmental networks, and social/behavioral relationships. Each category illustrates specific steps of the nominal data mining workflow, with an emphasis on handling missing values and exploring relationships among nominal labels. The datasets encompass a variety of bipartite network structures, i.e., weighted, unweighted, modular, and nested, demonstrating how the NIMAA pipeline supports pattern recognition, clustering, and predictive modeling across diverse domains.

### Proteomic data benchmarking

The bird’s-eye view of a dataset reflects the structure and distribution of missing values and can serve as an important metric for comparing the performance of different analytical tools. This is particularly relevant in proteomic datasets [26], where a wide variety of software platforms and search engines exist, each with numerous parameters, scoring algorithms, and tuning options. These variations make direct comparisons between tools inherently complex and often inconsistent. To address this challenge, we employ the initial steps of the NIMAA pipeline to assess and compare data completeness and structural integrity in protein quantification outputs by analyzing patterns of missingness. By visualizing and quantifying how missing values are distributed across bipartite representations of the data, NIMAA provides insights into tool-specific biases and structural distortions introduced during processing. Because all platforms are applied to the same underlying raw mass spectrometry data in this benchmarking setting, the differences discussed in this section should be interpreted primarily as software-specific processing effects rather than direct biological differences. We used WOMBAT-P ProteoBenchDDA v0.9.11 protein identification and quantification outputs to conduct a benchmarking pipeline using the NIMAA R package [27]. While this study focuses on DDA (data-dependent acquisition)-based proteomics data, which are known to exhibit structured missingness due to stochastic precursor selection, future work could extend the NIMAA framework to DIA (data-independent acquisition)-based datasets, where missingness patterns differ but still reflect underlying acquisition and quantification constraints. This framework allows us to evaluate how different quantification tools produce missing data systematically and to identify structural patterns that may affect downstream analyses. These standardized datasets include protein-level outputs from several established proteomics software platforms, including MaxQuant, CompOmics, Proline, and TPP (Trans-Proteomic Pipeline).

**Fig. 2.**
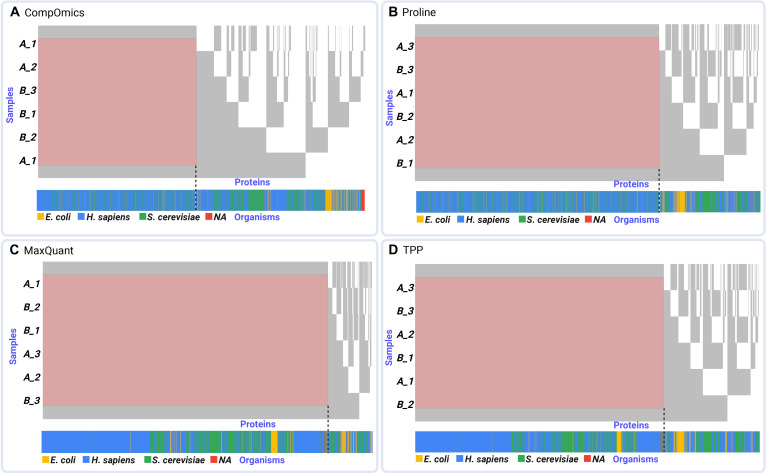
Bird’s-eye view of proteomics datasets. This figure shows the row-wise results of submatrix extraction using the “Rectangular_element_max” mode, applied to each dataset. The method identifies the largest rectangular area without missing values, highlighting the number of complete (non-NA [not available]) protein entries retained for each platform: (A) CompOmics, (B) Proline, (C) MaxQuant, and (D) TPP (Trans-Proteomic Pipeline). The heatmap plots next to each dataset represent the species categories, including *Homo sapiens* (blue), *Saccharomyces cerevisiae* (green), *Escherichia coli* (yellow), and NAs (red), which comprise UniParc accession numbers and mostly contain contaminant entries.

For consistency across platforms, we extracted columns corresponding to peptide quantification per sample, typically labeled as “number_of_peptides_[A/B]_X”. Each dataset contained varying numbers of quantified proteins across 2 conditions, with 3 replicates each. While the number of columns is fixed across all datasets, the number of quantified proteins (rows) varies. To retain as many proteins as possible while minimizing missing values, we applied the extractSubMatrix() function with the mode set to “Rectangular_element_max”. This method identifies the largest possible rectangular submatrix with complete data, maximizing the number of retained proteins (rows) across the fixed set of 6 samples (columns). Several metrics were then compared, including the size of the nonmissing submatrix, nestedness temperature score, relative nonmissingness (proportion of observed entries), and overall completeness (proportion of nonmissing values in the submatrix relative to the original dataset). These results are summarized in Tables 1 and 2, and the row-wise outcome of rectangular max has been visualized in Fig. 2.

**Table 1.** Summary comparison of 4 proteomics datasets in WOMBAT-P. The table presents the dataset size, the size of the largest complete submatrix, 2 proportions (a) total number of nonmissing values divided by total possible values (number of rows × 6), and (b) number of rows in the complete submatrix multiplied by 6, divided by total possible values, and the nestedness temperature scores.

Dataset name	No. of rows in initial dataset	Percentage of nonmissing values	Number of rows in the extracted complete submatrix	Proportion of extracted complete submatrix	Nestedness (temperature score)
CompOmics	5,862	0.446	2,612	0.671	23.276
MaxQuant	5,195	0.866	4,500	0.985	3.948
Proline	4,999	0.710	3,547	0.882	16.721
TPP	5,503	0.723	3,976	0.895	16.202

**Table 2.** Empirical permutation tests of organism-ordering randomness. Lower adjusted *P* values indicate that the observed order of organism labels is less random than expected under permutation, reflecting structured clustering. Consistent clustering in both complete and missing parts (as in MaxQuant and TPP) suggests systematic organization of quantification results, while a random pattern (as in Proline’s complete submatrix) indicates unbiased distribution after filtering.

Software	Matrix part	p_adj	Sig	Interpretation
CompOmics	Missing part	0.00027	***	Nonrandom (clustered)
CompOmics	Complete submatrix	0.0103	*	Nonrandom (clustered)
MaxQuant	Missing part	0.00027	***	Nonrandom (clustered)
MaxQuant	Complete submatrix	0.00027	***	Nonrandom (clustered)
Proline	Missing part	0.00027	***	Nonrandom (clustered)
Proline	Complete submatrix	0.215	ns	Indistinguishable from random
TPP	Missing part	0.00027	***	Nonrandom (clustered)
TPP	Complete submatrix	0.00027	***	Nonrandom (clustered)

**Fig. 3.**
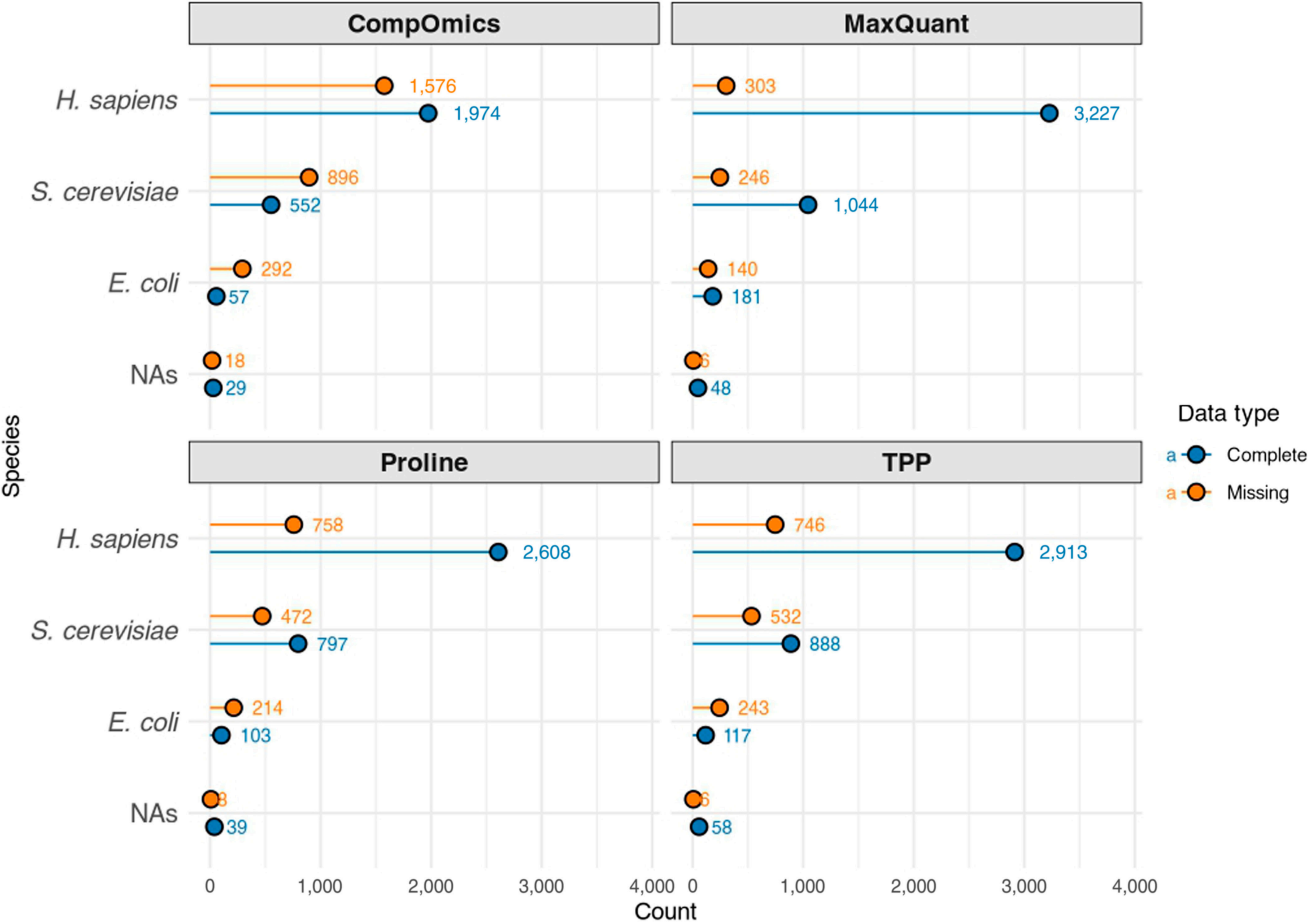
Species composition in complete and submatrices with missing values of identified proteins across proteomics software outputs. For each dataset generated by CompOmics, MaxQuant, Proline, and TPP (Trans-Proteomic Pipeline), the table reports the frequency of protein identifications per organism within the largest complete submatrix (nonmissing values) and in the complementary set containing missing values. Species categories include *Homo sapiens*, *Saccharomyces cerevisiae*, *Escherichia coli*, and NAs (not available), which comprise UniParc accession numbers and mostly contaminant entries.

MaxQuant demonstrated the highest proportion of extracted complete submatrix (98.5%) and percentage of nonmissing values (86.6%), indicating robust and consistent protein quantification. In contrast, CompOmics, although originally reporting the highest number of quantified proteins (5,862), retained the smallest nonmissing submatrix and exhibited the lowest nestedness, with a higher temperature score (23.276), suggesting more noise and random missingness. The TPP and Proline platforms showed intermediate performance. Here, performance refers to the ability of each platform to produce protein quantification data that is both complete and structurally coherent, as reflected by the size of the nonmissing submatrix, the organization of missing values measured by nestedness and temperature scores, and the biological plausibility of species-level clustering. These platforms had moderate-sized complete submatrices (72.3% and 71%, respectively) and better nestedness temperature scores, reflecting distinct data structures. These findings highlight platform-specific strengths: MaxQuant excels in producing more complete, hierarchically organized data with less noise, while CompOmics shows greater variability and weaker structural organization of missingness relative to the other platforms (Fig. 2). Importantly, these structural differences should not be interpreted as a formal classification of the missing-data mechanism (e.g., MAR, or MNAR), but rather as descriptive features of how missingness is organized across software outputs. The TPP and Proline outputs fall between these extremes. Such insights are valuable for guiding platform selection based on specific study goals, addressing an ongoing challenge in computational proteomics analysis. Importantly, in this benchmarking setting, all software tools were applied to the same underlying raw mass spectrometry data. Therefore, differences in missingness patterns across platforms do not reflect true biological absence of proteins or differences in detection limits between experiments but instead arise from tool-specific processing choices, filtering strategies, scoring functions, and identification thresholds. While species-level abundance differences in mixed-species samples provide a biological context, the observed variation in missingness structure primarily captures how different software implementations handle identical input data. In this sense, missing values serve as diagnostic indicators of computational behavior rather than direct biological signals. As shown in Fig. 2, this is evident when examining both complete submatrices and those containing missing values. In this figure, the bottom 4-color bar (red, blue, green, and gray) denotes proteins originating from *Escherichia coli*, *Saccharomyces cerevisiae*, *Homo sapiens*, and proteins of unknown origin, respectively. In the CompOmics and Proline outputs, these colors appear almost randomly distributed, suggesting minimal organism-specific clustering. In contrast, MaxQuant and TPP exhibit more distinct clustering patterns, with proteins from the same organism grouped more consistently, indicating a less random and more structured arrangement. Interestingly, we observed that among the benchmarked software packages, MaxQuant outperformed others in terms of the number of identified proteins in the complete submatrix *vs.* the submatrix with missing values across all organisms. For TPP and Proline in *E. coli* and for CompOmics in both *E. coli* and *S. cerevisiae*, the opposite trend was observed, with more proteins identified in the submatrix containing missing values (Fig. 3). Since human proteins are generally better annotated than those of *S. cerevisiae* or *E. coli*, these results indicate that MaxQuant is more effective at handling organisms with less comprehensive annotations, further underscoring its advantage across diverse species. Overall, this comparison underscores that evaluating proteomics software should go beyond simple counts of quantified proteins and instead incorporate completeness, structured missingness, and biological coherence as integrated measures of data quality.

In addition to completeness and nestedness, we assessed whether the distribution of organism labels within each dataset was random or exhibited clustering patterns using a permutation-based randomness test. For each dataset and matrix part, we compared the observed organism-label sequence against 5,000 random permutations of the same sequence, thereby preserving the marginal label frequencies while randomizing their order. Empirical *P* values were calculated from this permutation null distribution using run-based clustering statistics, and multiple testing correction was performed using the Benjamini–Hochberg false discovery rate procedure. This test evaluates whether the observed ordering is more structured than expected by chance under a null model of random label arrangement, without assuming that the observed sequence elements are independently generated. As summarized in Table 2, both MaxQuant and TPP showed significant nonrandom clustering (false discovery rate-adjusted *P* < 0.001) in both complete and missing-value regions, suggesting internally consistent organization and potentially more biologically coherent processing. CompOmics also displayed clustering, though with higher variability between matrix parts, whereas Proline’s complete submatrix was indistinguishable from random (p_adj ≈ 0.21), implying that its filtering step reduces organism-specific bias. Together, these results reinforce that the missingness structure itself encodes information about software-specific data organization.

Although the present example uses a small controlled benchmarking design, the intended application of NIMAA is not limited to software comparison. More broadly, the framework can be applied to larger biological and clinical proteomics datasets represented in bipartite form, where structured missingness may reflect technical, cohort-specific, or biologically meaningful patterns that complement downstream analysis.

While differences in missingness patterns across software platforms reflect a combination of computational, technical, and biological factors, this controlled setting illustrates how structured missingness can encode systematic signals. In contrast, in real-world datasets presented in subsequent sections, these patterns reflect biologically, ecologically, and behaviorally meaningful constraints, highlighting the broader applicability of this framework.

### Biomedical and pharmacological networks

We applied the NIMAA framework to 3 biomedical datasets, BeatAML, DrugComb, and Herb–Ingredient, covering complementary domains of drug response, combination therapy, and natural compound associations. These datasets vary substantially in scale and structure: BeatAML includes 122 inhibitors tested across 528 patient samples (~26% missingness); DrugComb integrates drug–cell line synergy measurements from large-scale screening studies with partially observed interaction matrices, and the Herb–Ingredient dataset comprises a filtered bipartite network of 51 ingredients and 28 herbs derived from an initial pool of 2,815 ingredients and 3,775 herbs. Together, these datasets provide diverse examples of nominal bipartite structures with varying degrees of sparsity and structured missingness, enabling systematic evaluation of the NIMAA workflow.

#### BeatAML dataset

**Fig. 4.**
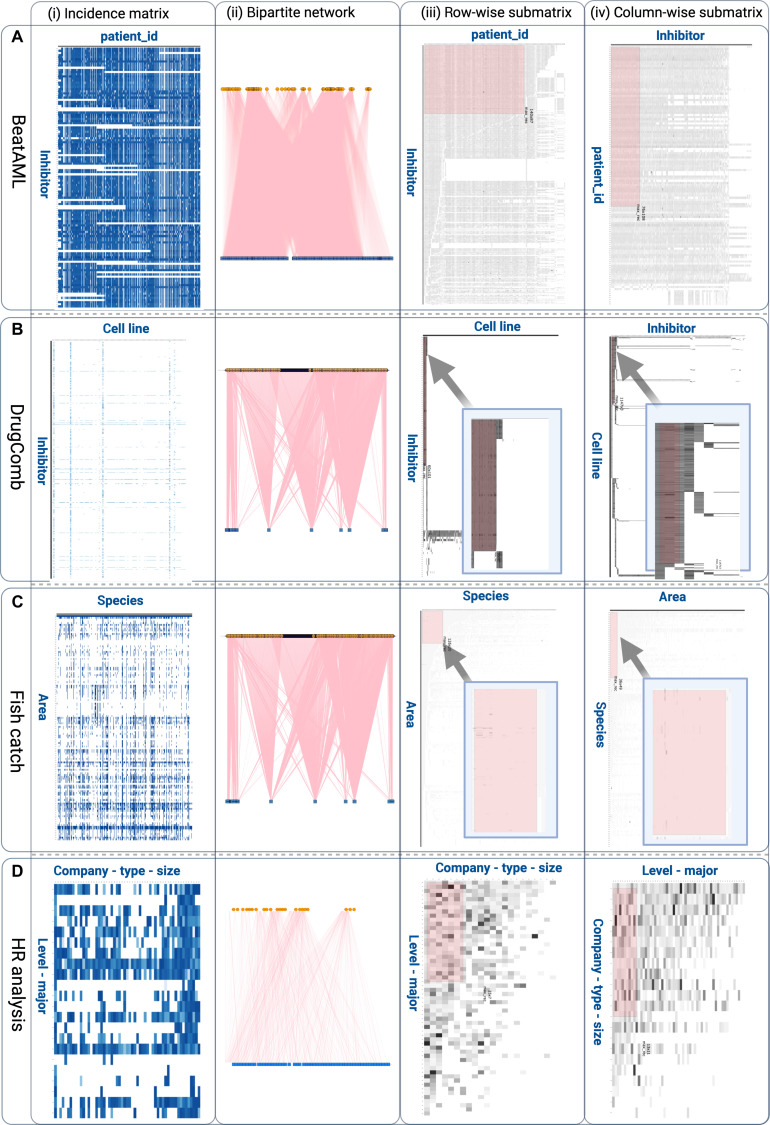
Visualizing data completeness and structure across diverse datasets using NomInal data Mining AnAlysis (NIMAA). For each dataset (A) BeatAML drug response, (B) DrugComb synergy analysis, (C) human resource (HR) analytics, and (D) fish catch records, the panels display (i) the incidence matrix highlighting the overall proportion of missing values, (ii) the corresponding bipartite network of nominal variables, (iii) the extracted row-wise submatrix with nonmissing values, and (iv) the extracted column-wise submatrix with nonmissing values. In the matrix representation, nonmissing values are shown as filled entries, while missing values (NAs [not available]) are represented by empty or uncolored cells, allowing direct visual assessment of the missingness structure.

The BeatAML dataset contains drug sensitivity measurements from *ex vivo* acute myeloid leukemia patient samples, offering a valuable resource for evaluating the efficacy of different inhibitors [28]. It comprises 122 distinct inhibitors tested across 528 patient samples and can be represented as a bipartite network, where the 2 nominal variables are inhibitors and patient identifiers. Edge weights correspond to median drug response values, providing a summary measure of drug sensitivity across patient samples. Analysis of the incidence matrix yields a nestedness temperature of 20.12, indicating a structured, nonrandom pattern of drug–patient associations. However, given the incomplete and uneven sampling of inhibitors across patient groups, this value likely reflects only a partial degree of nested organization relative to what might be expected in a more comprehensively measured dataset. The dataset contains 26% missing values, with the largest square complete submatrix covering 96 inhibitors and 96 patients (Fig. 4A). These missing values are not uniformly distributed; rather, their arrangement suggests structured missingness, supported by the observed nestedness pattern and the presence of a large complete submatrix, rather than being consistent with purely random absence.

Previous work by Jafari *et al.* [29] and Mirzaie *et al.* [30] demonstrated how leveraging the topology of missingness in such bipartite networks, specifically by examining the complement space of absent associations, can help select novel and clinically relevant drug combination strategies for acute myeloid leukemia. In this context, the missing value structure itself becomes an informative layer, guiding the identification of promising inhibitor–patient pairings for combination therapy design.

#### DrugComb dataset

The DrugComb dataset serves as a comprehensive resource that integrates cancer drug combination screening studies from various high-throughput experiments [31]. It provides data on synergistic and antagonistic effects of drug pairs tested across multiple cancer cell lines. In the NIMAA framework, this dataset is structured as a bipartite network in which the nominal variables represent drug combinations and corresponding cell line samples. The edge weights reflect the observed synergy scores, such as the Bliss or Loewe models. In the present analysis, the corresponding incidence matrix comprised 4,150 drug combinations across 112 cell lines, providing a large and sparse bipartite structure for evaluating missingness and projection-based clustering (Fig. 4B). This configuration facilitates the application of combinatorial effects and potential relationships between drug pairs and specific cancer types (see the NIMAA Shiny app for details). The synergy values in this dataset, which contains missing entries, are analyzed using the NIMAA framework, including clustering and projection-based methods, to uncover latent patterns and structure in the data and to prioritize candidate drug combinations for further investigation. The resulting structures are used to generate a ranking of candidate combinations, supporting the detection of potential therapeutic synergies and cell line–specific responses.

#### Herb–Ingredient dataset

The second version of the TCMID (Traditional Chinese Medicine Integrated Database) is a key resource in the field of Traditional Chinese Medicine (TCM) herbs [32]. TCMID provided a comprehensive resource by incorporating ingredient-specific experimental data obtained from herbal mass spectrometry spectra. This extensive dataset encompassed 2,815 ingredients and 3,775 herbs. To ensure an adequate amount of data, a filtering criterion was applied, retaining only those herbs that contained a minimum of 40 ingredients and ingredients that were present in at least 40 herbs. Consequently, a dataset comprising 51 ingredients and 28 herbs was obtained (see the NIMAA Shiny app for details). This heterogeneous nature of herbal formulations suggests that ingredient sharing across herbs may be driven more by empirical tradition than systematic design.

Building on such ingredient–herb associations, earlier work by Jafari *et al.* [33] demonstrated how bipartite network analysis of ingredient–herb relationships in TCM can reveal molecular patterns underlying traditional classifications, such as meridians and properties. In this context, the alignment between ingredient clusters and classification profiles becomes an informative layer, bridging empirical TCM knowledge with modern chemical and molecular data to support phenotype-based drug discovery.

### Ecological and environmental networks

To demonstrate NIMAA’s applicability beyond biomedical contexts, we analyzed 2 ecological datasets: the historical Robertson plant–pollinator network and the fish catch annual dataset. The Robertson dataset, originally comprising 1,429 insect species and 456 plant species, was filtered to a network of 122 pollinators and 24 plants to ensure sufficient interaction density. The fish catch dataset captures species–area relationships across multiple years (2009 to 2014), with structured absence patterns. These datasets illustrate how structured missing values can capture ecological specialization, temporal dynamics, and environmental constraints, providing insights beyond observed interactions.

#### Robertson dataset

Charles Robertson’s historical plant–pollinator dataset provides a unique opportunity to study the structure of missing interactions over an extended time scale. Collected between 1884 and 1916 in Macoupin County, Illinois, USA, the data originally recorded 1,429 insect species visiting 456 plant species. When represented as a bipartite incidence matrix, missing values correspond to unobserved or absent interactions between specific plant–pollinator pairs [34].

To improve interpretability, we applied a filtering step, retaining only insects that visited at least 30 flowers and plants visited by at least 50 insects. This yielded a reduced network of 122 pollinator species and 24 plant species. The resulting matrix reveals distinct patterns of missingness: Clusters of complete interaction data for some pollinator–plant groups coexist with large, structured gaps for others (see the NIMAA Shiny app for details). For example, 8 pollinator species form complete interaction blocks with 8 plant species (specialized relationships), while 24 pollinators interact with only 4 plants, generating a more sparsely populated yet patterned submatrix (generalized relationships). These nonrandom missingness structures are consistent with well-established ecological patterns, such as species specialization, seasonal overlap, and spatial constraints, which are known to shape plant–pollinator interaction networks, rather than arising solely from random sampling absence [19,35,36]. This interpretation is further supported by prior ecological studies showing that interaction sparsity and block patterns in bipartite networks often reflect functional roles and community organization in complex ecosystems.

#### Fish catch annual dataset

The fish catch annual dataset documents annual fish and shellfish catches in the Northeast Atlantic across multiple decades, where rows represent species and columns represent areas. Missing values in the bipartite incidence matrix correspond to zero-reported or unrecorded catches for certain area–species combinations over 6 years (2009 to 2014). Visual inspection and nestedness analysis, with the temperature score of 2.66, indicate a highly nested structure, revealing that these gaps are not uniformly distributed: Some species exhibit continuous reporting across years in specific areas, while others display patchy but temporally clustered absences. This pronounced nested structure suggests that missingness is strongly organized rather than random. Such structured missingness may reflect historical changes in fishing effort [37], climate change-related species availability [38], or regulatory mechanisms [39], although these represent potential explanatory hypotheses rather than directly inferred causes.

By projecting the network and clustering species or years, NIMAA reveals covarying groups where missing values align across blocks (Fig. 4C, incidence matrix). These patterns indicate structured covariation in the data and may be consistent with shared ecological or management influences, although such interpretations require further domain-specific validation [39]. This pronounced block-structured patterning provides additional insight into long-term biodiversity dynamics and fishing practices, reinforcing the importance of interpreting the missingness form alongside observed values in sustainability analyses.

### Human behavior and social systems

We further applied NIMAA to datasets from human resources, online education, and retail transactions, demonstrating its versatility across social and commercial domains. These datasets are characterized by high sparsity and structured absence patterns, where missing values correspond to unobserved interactions such as unrecorded performance metrics, inactive student–topic pairs, or nonpurchased product–customer combinations. Despite differences in scale and context, all 3 datasets exhibit block-like missingness patterns that reveal subgroup structures and behavioral heterogeneity, highlighting the potential of NIMAA to extract meaningful signals from incomplete nominal data.

#### Human resource analytics dataset

In the human resource analytics dataset, employees and organizational attributes (e.g., department, job satisfaction, and promotion status) form the 2 nominal variable sets. For the selected representation used here, the incidence matrix consisted of 22 employee-level-major groups by 57 company-size categories, enabling the inspection of structured sparsity and subgroup organization. Missing entries, such as absent performance ratings, do not occur randomly but often cluster within specific employee groups or organizational units. This block-like missingness may arise from policy differences, survey nonresponse patterns, or role-specific reporting gaps.

When visualized as an incidence matrix, these missing clusters reveal structured subgroup patterns within the data (Fig. 4D). Although this example is primarily illustrative, the observed block-like sparsity pattern provides a compact structural summary of the organization of missing associations in this nominal dataset. Such patterns may reflect underlying organizational or behavioral factors, although their specific interpretation would require additional contextual or metadata-driven validation. Instead of immediate imputation, NIMAA first leverages these patterns to guide clustering, revealing associations between structured missingness and turnover risk. This approach ensures that missing values contribute to the understanding of organizational behavior rather than being treated solely as noise.

#### Student discussion dataset

The student discussion dataset models students and discussion topics from an e-learning platform as a bipartite graph. In the selected incidence matrix, 27 student nationality–gender groups were related to 38 grade–topic categories, yielding a sparse nominal structure suitable for examining topic-specific absence patterns. Missing values correspond to unengaged topic–student pairs. Far from being random, these gaps often form topic-specific blocks, where certain groups of students consistently avoid or remain inactive in particular threads. Such structured absence patterns can indicate topical disengagement, prerequisite knowledge gaps, or social clustering in participation.

By analyzing the topology of these gaps before imputation, NIMAA helps identify passive learner communities and isolated topics, allowing targeted interventions (see the NIMAA Shiny app for details). Structured missingness here becomes an educational signal, enabling the detection of learning bottlenecks and informing personalized course design.

#### Online retail dataset

Here in the retail transaction dataset, customers and products form a bipartite network where edge weights reflect purchase volume or value. Missing values dominate the incidence matrix, representing customer–product pairs with no recorded purchases. These absences are not uniformly distributed: Certain product groups show high copurchase coverage among specific customer clusters, while others are completely absent for those same groups, forming structured void regions in the matrix.

Such patterns often reflect product specialization, seasonality, or targeted marketing effects. NIMAA’s analysis reveals how these structured absences can complement purchase data to refine customer segmentation and product recommendation strategies (see the NIMAA Shiny app for details). By preserving the shape of missingness during early structural analysis, NIMAA can help reveal subgroup-specific gaps and block-wise customer–product patterns that may inform downstream tasks such as customer segmentation, demand exploration, or recommendation modeling.

## Conclusion

This work reframes missing value forms in nominal datasets from being mere analytical hindrances to becoming informative structural features. By modeling data as bipartite networks and examining the form, distribution, and topology of missingness, the NIMAA framework reveals that gaps often encode latent biological constraints, operational biases, or domain-specific organization. Across proteomics benchmarking, pharmacogenomics, ecological surveys, and social systems, we demonstrate that structured missingness can illuminate system modularity, nestedness, and category-level similarities, often in ways that enhance interpretability beyond what observed values alone can provide. Rather than defaulting to early imputation or exclusion, our approach prioritizes the analysis of missingness structure before any value inference, using optional imputation only as a secondary step to assess the stability of the identified patterns. We note that the NIMAA framework does not aim to formally distinguish between missingness mechanisms such as MAR or MNAR. Instead, it focuses on identifying structured patterns in missing data. While such patterns may be consistent with specific mechanisms, their interpretation remains hypothesis-generating and requires further validation. Future work could incorporate simulation studies with controlled missingness mechanisms to systematically evaluate the sensitivity and limitations of the approach. It would also benefit from systematic benchmarking against standard missing-data approaches using controlled simulations and reference datasets to further quantify the added value of structural missingness analysis. In addition, future extensions should benchmark structural metrics such as nestedness and modularity against appropriate bipartite null models, for example, degree-preserving randomizations, to determine whether the observed patterns exceed those expected from sparsity structure alone. At the same time, the current framework has several practical limitations. Its performance and interpretability may be influenced by dataset size and sparsity, the choice of filtering or submatrix extraction thresholds, and the granularity at which nominal variables are defined. These factors should be considered carefully, particularly when applying NIMAA to very large, highly sparse, or heterogeneous datasets. These findings advocate for a paradigm shift: Missing values should be treated not simply as noise to be corrected but as valuable signals capable of enriching discovery and guiding more context-aware modeling in complex systems.

## Data Availability

All data sources used in this study are linked to their corresponding panels in the NIMAA Shiny web application, available at https://ehsan-zangene.shinyapps.io/nimaa_app/, allowing direct access to the datasets used in the case studies. The full analysis codes are publicly available at https://github.com/esnzgn/NIMAA_cases. In particular, the benchmarking code and high-resolution figure outputs for the NIMAA mass spectrometry proteomics analysis are available at https://github.com/esnzgn/NIMAA_MS_bench. The NIMAA package is also actively maintained and publicly available through CRAN.
